# A discussion paper on stigmatizing features of diabetes

**DOI:** 10.1002/nop2.112

**Published:** 2018-01-24

**Authors:** Samereh Abdoli, Mehri Doosti Irani, Lynda R. Hardy, Martha Funnell

**Affiliations:** ^1^ College of Nursing University of Tennessee Knoxville TN USA; ^2^ Shahr‐e‐Kourd University of Medical Sciences Shahr‐e‐Kourd Iran; ^3^ Department of Learning Health Sciences University of Michigan Medical School Ann Arbor MI USA

**Keywords:** diabetes, diabetes‐related stigma, stigma

## Abstract

**Aim:**

This manuscript aims to describe stigmatizing features of diabetes.

**Design:**

This article presents a narrative review of literature pertaining to stigma surrounding diabetes in different contexts.

**Methods:**

A literature search was conducted in CINAHL, PubMed and Web of Science for qualitative studies published between 2007–2017. The search was completed using various combinations of diabetes, T1DM, T2DM, stigma, social/public stigma, internalized/self‐stigma, stigmatization and diabetes‐related stigma in English. The reviewers then independently reviewed the eligible studies (*N *= 18) to extract data.

**Results:**

From the 18 studies included in this narrative review, seven features related to stigma in diabetes were identified. People with diabetes were most notably considered and stigmatized as being “sick,” “death reminder,” “rejected marriage candidate,” “self‐inflicting,” “contagiousness,” “requiring a dietary modification” and “drunk or drug abuser.”

## 1 INTRODUCTION

Diabetes, a major chronic health condition, is increasing healthcare challenges nationally and globally. It is estimated that, globally, 387 million people have diabetes; this number is expected to rise to 592 million by 2035 (International Diabetes Federation, n.d.). In response to a world pandemic of diabetes, it is crucial to consider that people living with diabetes are stigmatized by their illness (Abdoli, Ashktorab, Ahmadi, Parvizy, & Dunning, 2014; Anderson‐Lister & Treharne, 2014; Hapunda, Abubakar, Van de Vijver, & Pouwer, 2015; Kato, Takada, & Hashimoto, 2014; Schabert, Browne, Mosely, & Speight, 2013; Singh, Cinnirella, & Bradley, 2012), especially those living with T1DM (Abu Hassan et al., 2013; Jaacks, Liu, Ji, & Mayer‐Davis, 2015; Spencer, Cooper, & Milton, 2014; Vishwanath, 2014).

Stigma, a discrediting attribute minimizing a person's value, is a multi‐dimensional construct including interpersonal and intrapersonal experiences (Goffman, 1963). It is defined as discriminatory behaviours directed towards people with the stigmatized condition (Bogart et al., 2008), although it is not limited only to the behaviours. Weiss, Ramakrishna, and Somma (2006) have suggested stigma is typically a social process, experienced or anticipated, characterized by exclusion, rejection, blame or devaluation that result from experience, perception or reasonable anticipation of an adverse social judgement about a person or group. This judgement is based on an enduring feature of identity conferred by a health problem or health‐related condition, and the judgement is in some essential way medically unwarranted (p.279).

### 1.1 Background

Stigma in diabetes contributes to a hidden burden of the chronic condition affecting multiple aspects of life of those with diabetes (Abdoli, Abazari, & Mardanian, 2013; Benedetti, 2014; Broom & Whittaker, 2004; Nicolucci et al., 2013). Diabetes‐related stigma may preclude diabetes management (Salamon, Hains, Fleischman, Davies, & Kichler, 2010), diabetes adherence (Mulvaney et al., 2011), multiple daily injections, participation in research studies, general health‐seeking behaviours (DiZazzo‐Miller et al., 2017; Jaacks et al., 2015) and insulin injections in unsanitary places (Abdoli, Doosti Irani, Parvizi, Seyed Fatemi, & Amini, 2013; Browne, Ventura, Mosely, & Speight, 2014; Shiu, Kwan, & Wong, 2003). In general, stigma may make individuals with diabetes frustrated by feeling different (Nurmi & Stieber‐Rodger, 2012; Vermeire et al., 2007), keeping their diabetes a secret, avoiding self‐management activities and seeking health‐promotion choices (Abdoli, Doosti Irani et al., 2013; Elissa, Bratt, Axelsson, Khatib, & Sparud‐Lundin, 2016; Fritz et al., 2016). This can place them at a higher risk for poor diabetes management and high prevalence of acute and chronic diabetes complications (Abdoli, Abazari et al., 2013; Browne et al., 2014).

Stigma in chronic illnesses such as HIV/AIDS has received considerable attention, but there has been limited attention given to stigma and diabetes (Browne et al., 2014). A small body of research exists related to understanding stigma as a social construct in different cultures. Culture affects how people exhibit alternate thinking, feeling and behaving processes that may affect stigmatization and discrimination towards people with diabetes. Such differences may affect the definition and manifestation of stigma (Weiss et al., 2006). A comprehensive understanding of stigma surrounding diabetes is important for informing policy and practice to improve the quality of care and quality of life for those living with diabetes (Schabert et al., 2013).

The literature review about stigma in diabetes aimed to describe stigmatizing features of diabetes in different countries around the world. The review of findings may provide a foundation for future research related to stigmatization in living with diabetes.

## 2 THE STUDY

### 2.1 Design

This article presents a narrative review of literature related to stigma in diabetes.

### 2.2 Method

#### 2.2.1 Search strategy

Each search was completed using various combinations of these search words: diabetes, T1DM, T2DM, stigma, social/public stigma, internalized/self‐stigma, stigmatization and diabetes‐related stigma. An electronic search of CINAHL, PubMed and Web of Science was conducted by two reviewers (S.A. and M.D.I.) to identify manuscripts published between 2007–2017 on diabetes and stigma.

#### 2.2.2 Inclusion criteria

**Figure 1:**
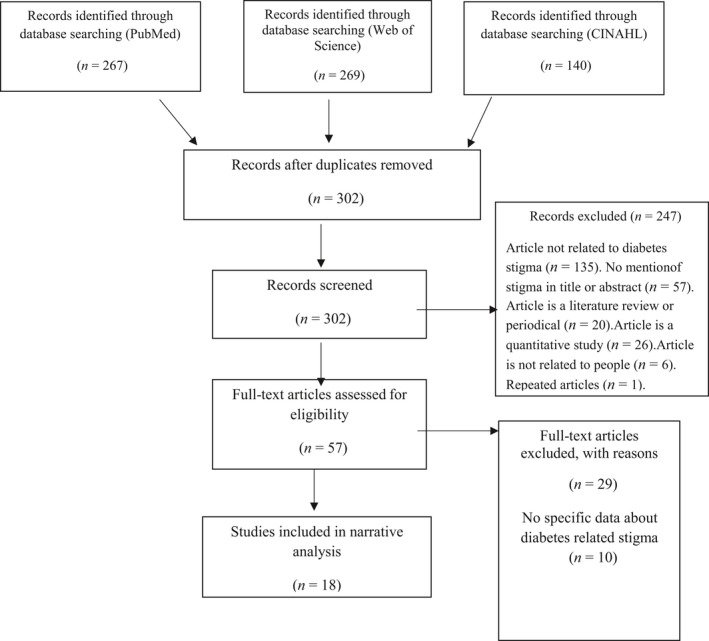
PRISMA flow diagram for manuscript related to stigma in diabetes

Qualitative studies were included in this review of literature. Articles had to focus on stigmatization against people diagnosed with T1DM, T2DM or both. Studies describing the stigmatized perception of people without diabetes towards those living with diabetes also were included. Studies that were excluded were not peer‐reviewed, did not provide enough information about stigmatized features of diabetes or described insufficient data related to stigma in diabetes for data extraction. Nineteen qualitative manuscripts were identified for inclusion in the review. Figure 1 shows the PRISMA flow diagram of this review.

#### 2.2.3 Data extraction

Two reviewers (S.A. and M.D.I.) evaluated abstracts to identify articles meeting the inclusion criteria. Then, eligible studies and full text of relevant articles to stigma in diabetes were carefully read by each reviewer independently. A data extraction form was adapted from the literature. Discrepancies between the two reviewers in the extracted data were resolved in consensus discussion.

#### 2.2.4 Ethical statement

The research team comprehensively reviewed all the relevant work and judged research quality and relevance. All the references also were acknowledged and fully cited.

## 3 RESULTS

Description of studies: Eighteen qualitative studies were analysed in this narrative review. Ten studies included T1DM participants, eight studies included T2DM and four studies included participants without diabetes. Five studies were conducted in an Asian population; two studies in Africa; and the remaining studies were conducted in the United States, Australia and the UK. Study characteristics can be found in Table 1.

**Table 1:** Included studies related to stigma in diabetes

Authors	Study design	Samples population	Sample size	Study setting
Abdoli, Abazari et al., (2013)	Content analysis	Adults with T1DM and without diabetes	26	Iran
Abdoli, Doosti Irani et al., (2013)	Content analysis	Adults with T1DM	33	Iran
Alzubaidi, McMamara, Chapman, Stevenson, and Marriott (2015)	Content analysis	Arab and Caucasian adults with T2DM	100	Australia
Browne et al. (2013)	Content analysis	Adults with T2DM	25	Australia
Browne et al. (2014)	Content analysis	Adults with T1DM	27	Australia
DiZazzo‐Miller et al. (2017)	Content analysis	Arab American healthcare providers	8	USA
Elissa et al. (2016)	Content analysis	Children with T1DM	10	Palestine
Hallgren, McElfish, and Rubon‐Chutaro (2015)	Content analysis	Marshallese with T2DM	15	USA
Hapunda et al. (2015)	Content analysis	Adolescence with T1DM, caregivers, healthcare providers	22	Zambia
Haugvik et al. (2016)	Content analysis	Children with T1DM, parents and endocrinologist	41	Tajikistan
Lin et al. (2008)	Content analysis	Adults with T2DM	41	Taiwan
Mendenhall and Norris (2015)	Content analysis	Adults with T2DM	27	Soweto
Singh et al. (2012)	Content analysis	Adults with diabetes	20	UK
Verloo et al. (2016)	Content analysis	Children with T1DM and parents	11	India
Vishwanath (2014)	Content analysis	T1DM	N/A	USA
Weiler (2007)	Content analysis	Latino adults with T2DM	10	USA
Weiler and Crist (2009)	Content analysis	Latino adults with T2DM	10	USA
Willig et al. (2014)	Content analysis	African American	35	USA
Winkley et al. (2015)	Content analysis	Adults with T2DM	30	UK

The literature review highlighted that diabetes‐related stigma is a complex issue. Some themes are interrelated and could not be separated. In these manuscripts, people with diabetes were mostly stigmatized as “sick and disabled,” “death reminder,” “rejected marriage candidate,” “self‐inflicting,” “contagious,” “requiring dietary modification” and “drunk or drug abuser.”

### 3.1 Sick

Seven studies in different countries (the United States, Canada, Australia, India, Iran and Palestine) reported that people with diabetes are stigmatized as being sick. The designation of “being sick” affects an individual's ability to experience a normal independent life, and is a common diabetes‐related stigma in Australia (Browne et al., 2014). One study in Iran found that young adults with diabetes perceive the social stigma of diabetes as being sick and disabled (Abdoli, Abazari et al., 2013). A similar result was found in Palestinian children with T1DM, who perceived diabetes as a stigmatizing condition that spoiled their identity as a healthy individual, making them feel like an outsider and not a normal person (Elissa et al., 2016). A study performed in a U.S. Arab American community found that individuals often viewed diabetes as a weakness or breakdown (DiZazzo‐Miller et al., 2017). Indian mothers of children with diabetes experienced diabetes‐related stigma when other people labelled their child as a “sick kid” (Verloo, Meenakumari, Abraham, & Malarvizhi, 2016). This finding is similar to Weiler's (2007) study and Weiler and Crist's (2009) study where Mexican American participants with diabetes experienced stigmatization as “being sick” and referred to the stigma as “The Big D.”

### 3.2 Death reminder

In three studies (Tajikistan, Iran and Soweto), individuals with diabetes were stigmatized as a “death reminder.” Being a “death reminder” has a strong connection of being stigmatized as “sick.” Children with T1DM in Tajikestan described their experiences of how people predict their premature death by saying, “You are very sick! You will die soon; you will not have a long life” (Haugvik, Beran, Klassen, Hussain, & Haaland, 2016), which is similar to (Abdoli, Abazari et al., 2013) in Iran. Some participants in Mendenhall and Norris's (2015) study also indicated how some people feel diabetes is a “death panel” by whispering about amputations due to diabetes and negative stories surrounding diabetes.

### 3.3 Marriage rejected candidate

Diabetes‐related stigmatization is considerably greater for younger, unmarried women, particularly in Asian countries. Delayed marriage is reported in people with diabetes in different countries such as Iran and India (Abdoli, Abazari et al., 2013). Iranians believe that women with diabetes are not suitable candidates for marriage due to high‐risk pregnancies, the potential of having a child with diabetes, and the role of a woman in the Iranian family (Abdoli, Doosti Irani et al., 2013). In a similar study in the UK, the South Asian community described public perception that views diabetes as a sign of physical inadequacy to traditional marriage (Singh et al., 2012). An unmarried Arab male in Australia described diabetes as a “disaster,” which makes both males and females with diabetes less desirable candidates for marriage due to a perceived connection between diabetes, erectile dysfunction and the passing of diabetes to their children (Abouzeid, Philpot, Janus, Coates, & Dunbar, 2013).

Marriage in India is a source of stress for individuals with diabetes and their families. Some Indian adolescents, especially girls with diabetes, experienced social stigmatization and were not wanted for marriage (Hapunda et al., 2015). This is also true for Indian mothers, who consider diabetes as a barrier for their daughters getting married (Verloo et al., 2016). Individuals with diabetes in London are thought to be unable to conceive or to have a normal pregnancy (Winkley et al., 2015).

A 2014 Australian study noted that participants experienced the termination (or threat of termination) of a romantic relationship due to diabetes. Fear of the negative impact of diabetes on their relationship was one of the main reasons highlighted by participants. They were worried about disclosing their diabetes to their partners or potential partners. It also was mentioned as a marriage barrier by some participants (Browne et al., 2014).

### 3.4 Self‐inflicting

Nine studies have noted that the community's perception about the cause and nature of diabetes can be stigmatizing. In several countries such as Iran (Abdoli, Abazari et al., 2013), Australia (Browne, Ventura, Mosely, & Speight, 2013), Taiwan (Lin, Anderson, Hagerty, & Lee, 2008), Ireland (Balfe et al., 2013) and the United States (Vishwanath, 2014), individuals with diabetes are considered to be self‐inflicting the disease. There are two common beliefs about diabetes that can be stigmatizing for people with diabetes: (i) diabetes is an illness of over‐indulgence with food (Lin et al., 2008) and (ii) diabetes is a result of an individual's own actions (Browne et al., 2013; Vishwanath, 2014). For example, the findings of Vishwanath's (2014) U.S. study suggested that most participants described diabetes as a disease that affects children who are lazy, unhealthy, fat, obese, lacking exercise and having an eating disorder (p. 516).

Overweight people, particularly in T2DM, are stigmatized for getting diabetes because of their lack of self‐control. In some cultures such as Hispanic or Latino, diabetes is seen as a punishment from God. Weiler (2007) wrote that the punishment ideology imposed a self‐associated stigmatization, which is similar to the Abdoli, Doosti Irani et al., study (2013) in Iran and the Browne et al. (2014) study in Australia.

### 3.5 Drink or drug abuser

Social stigma attached to insulin injections as a form of drug abuse is another important feature of stigmatization in living with diabetes (Berlin, Sass, Davies, Reupert, & Hains, 2005). For example, negative social connotations about insulin injections are seen in different countries (Brod, Kongsø, Lessard, & Christensen, 2009; Buchbinder et al., 2005). Insulin injections can be misunderstood as drug abuse in Iran (Abdoli, Doosti Irani et al., 2013), Taiwan (Chen, Tseng, Huang, & Chuang, 2012; Lin et al., 2008) and Australia (Browne et al., 2014). Tajukestani's children expressed being stigmatized as drug abusers while trying to inject insulin in public places (Haugvik et al., 2016). Australian participants also described being worried about, or having experienced, being mistaken for a drug abuser while injecting insulin. This was particularly the case for those who injected insulin with a vial and a syringe before the advancement of insulin pens and pumps (Browne et al., 2014). Participants with T2DM in Kuala Lumpur also expressed their feelings of stigmatization as a barrier for insulin injection, which can be misunderstood or stigmatized as drug abuse (Abu Hassan et al., 2013).

### 3.6 Requiring dietary modification

Reviewed articles referred to the stigmatization of people with diabetes due to life modifications, especially dietary modifications and restrictions. The required treatment regimen for diabetes management includes actions that often are noticeable by others. This includes eating at specified times, which may be associated with some degree of stigma (Chatterjee & Biswas, 2013; Fukunaga, Uehara, & Tom, 2011). For example, in the United States, African‐American women in the Willig, Richardson, Agne, and Cherrington (2014) expressed their frustration towards family and friends who make decisions for them about what they can eat. Iranian participants also pointed to the stigma of people with diabetes because of dietary restrictions (Abdoli, Doosti Irani et al., 2013).

### 3.7 Having a contagious disease

A few of the reviewed articles indicated people without diabetes may stigmatize those living with diabetes as being contagious. For example, the Lin et al. (2008) study on Taiwanese individuals with T2DM found that some people believe diabetes is an infectious disease, and they stigmatize people with diabetes as contagious. Hapunda et al. (2015) noted that in Zambia there is a fear of getting diabetes in a social setting. Therefore, some children who participated in a study mentioned that the community perceived them as “infectious” and some of their peers would deny playing with them because they may catch diabetes (Hapunda et al., 2015).

### 3.8 Limitations

The limitation of this manuscript is having a retrospective review of previously published manuscripts chosen at the authors' discretion and selected electronic databases.

## 4 DISCUSSION

Individuals with diabetes are stigmatized as sick and disabled (Browne et al., 2014; Weiler, 2007), which can be the underlying foundation of most of the stigma surrounding diabetes (Shestak, 2001; Weiler & Crist, 2009). Being stigmatized as sick and disabled is itself a stigma in some cultures (Kesavadev et al., 2014). This feature of stigma has the ability to make people dependent on others throughout their life and impose a financial burden on family and society (Abdoli, 2011). It also leads to a greater burden for people with diabetes in certain population sub‐groups such as young adults and women, particularly in Asian countries (Abdoli, Abazari et al., 2013; Doosti Irani, 2014). Some Asian countries view diabetes as a sign of physical inadequacy rooted in being sick and disabled. This perspective leads to a disproportionate burden of diabetes on young adults, particularly women, and affects their marriage potential (Ahmadi, MaslakPak, Anoosheh, Hajizadeh, & Rajab, 2009; Maslakpak, Anoosheh, Fazlollah, & Ebrahim, 2010; Patel, Eborall, Khunti, Davies, & Stone, 2011). People in Asian countries assume that those living with diabetes cannot perform duties as a mother or as a marital partner as they are considered “sick and disabled” (Abdoli, Doosti Irani et al., 2013). Individuals with or without diabetes think that women with diabetes are infertile or at a high risk for pregnancy (Abdoli, 2011). Women are thought to transmit diabetes to their child, who will inevitably suffer foetal death or be born with other congenital disorders. Men are considered to be sexually dysfunctional due to diabetic impotency. The financial burden of diabetes medication and associated complications is of great concern to men and women affected with diabetes (Browne et al., 2014).

Even in the 21st century, communities are not aware of diabetes aetiology and some consider diabetes a punishment or a result of one's lack of self‐control (Browne et al., 2013; Caban, & Walker, 2006; Hjelm, Bard, Nyberg, & Apelqvist, 2003; Lin et al., 2008; Vishwanath, 2014). Individuals also do not feel safe to inject insulin in public places because they might be misunderstood as a drug abuser or drunk while they are experiencing symptoms of hypoglycaemia (Abdoli, Doosti Irani et al., 2013; Browne et al., 2014; Ho & James, 2006; Lin et al., 2008).

## 5 CONCLUSION

This review of articles indicates the issue of stigmatization for people with diabetes has been an ongoing significant psychosocial issue associated with diabetes globally. Although an increasing number of declarations and laws are aimed at health equality of people with diabetes, discrimination and stigmatization is still broadly diffused (Benedetti, 2014). The review highlighted misconceptions and negative or exaggerated beliefs about diabetes in different countries continue and must be addressed to end diabetes‐related stigma. Creating a world knowledgeable about diabetes would alleviate the stigma surrounding diabetes in different cultures. Healthcare professionals, especially those working with people with diabetes, must consider strategic and worldwide policies including community education, family education and education for healthcare providers as a core component in all destigmatizing programmes and activities. It is also necessary to discuss stigma and help individuals identify strategies addressing stigma related to diabetes and the pivotal role of individual involvement in advocacy and policy efforts related to diabetes.

Diabetes self‐management education requires addressing the stigma while trying to empower those living with diabetes, particularly in Asian, Middle Eastern and African‐American communities.

## CONFLICT Of INTEREST

The authors do not have any conflict of interest.

## AUTHOR CONTRIBUTIONS

AS: Data collection; AS, MDI: Data analysis; AS, MDI: Manuscript writing; HL, MF: Critical revisions for important intellectual content.
